# Measuring Thickness of Middle Ear Mucosa Using MRI and CT Imaging versus Histopathology

**DOI:** 10.1155/2012/962496

**Published:** 2012-03-08

**Authors:** Mary Ann Nyc, Sang Gyoon Kim, Anil Kapoor, Timothy Jung

**Affiliations:** ^1^Department of Otolaryngology and Head and Neck Surgery, Loma Linda University School of Medicine, Loma Linda, CA 92354, USA; ^2^Otolaryngology Research, Jerry L. Pettis Memorial Veterans Administration Medical Center, Loma Linda, CA 92357, USA; ^3^Mineral Metabolism Research, Jerry L. Pettis Memorial Veterans Administration Medical Center, Loma Linda, CA 92357, USA

## Abstract

*Objective*. Otitis media (OM) is characterized by increased middle ear effusion and inflammation of middle ear tissue. In this study, we compared two radiographic methods of analyzing inflammation by measuring mucosal thickness (MT). *Methods*. 28 chinchillas were divided into three treatment groups consisting of a vehicle control group and two glucocorticoid groups. 6 underwent treatment by vehicle control, 10 were treated with ciprofloxacin 0.3%/dexamethasone 0.1% (DEX), and 10 received ciprofloxacin 0.2%/hydrocortisone 1% (HC). 96 hrs post-LPS inoculation, chinchillas were euthanized and their temporal bones were removed for analyses. *Results*. MRI scans (*F* = 146.0861, *P*-value <0.0001) and histology (*χ* ^2^ = 40.5267, *P*-value <0.0001) revealed statistically significant differences in MT measurements among treatment groups, whereas CT imaging did not. DEX-treated chinchillas exhibited overall significantly smaller MT values. *Conclusion*. Imaging MT was effective for determining severity of inflammation due to OM. Previous gold standard methods using histopathology compromise tissue integrity by chemical manipulation and dehydration effects. MRI and CT scanning are viable tools to preserve tissue and examine changes in MT. In this study, MRI provided more information about internal, soft tissue structures. In a clinical setting, MRI could be used for diagnosing and tracking severe or chronic OM.

## 1. Introduction

Considerable variation exists in the clinical diagnosis of otitis media (OM). For the majority of clinicians, successfully diagnosing OM is achieved from accurate otoscopic examination in which bulging of the tympanic membrane is evidenced along with pervasive erythema around the ear canal [1]. Traditionally, the causes of OM are classified as eustachian tube dysfunction or infection [2]. Consistent with findings of infection, increased middle ear effusion (MEE) coupled with inflammation of middle ear mucosa and increase of mucosal thickness (MT) take place as part of the body's immune response to foreign pathogens. Unchecked, misdiagnosed, or inappropriately treated OM can persist chronically and lead to complications. In order to control and prevent OM progression, some imaging modalities have been used to diagnose and followup with treatment progression. Though there are many imaging tools and few guidelines for their use, it does seem that anatomic imaging vis-a-vis CT or MRI is frequently favored.

In the scientific literature, MRI is often used when more accurate views of soft tissue are needed [3, 4]. Conversely, CT is used to show the extent of bony changes [4]. In terms of severe OM cases, CT use is important to identifying early stages of disease and for assessing progression that involves bony regions. Though MRI and CT seem to have clear delineations for their use, they are frequently used concurrently and interchangeably.

Therefore, the aim of this study was two fold:

1. to compare two glucocorticoid treatments in order to evaluate drug effectiveness in reducing overall MT,
2. to evaluate and compare the use of radiographic tools (MRI and CT) in visualizing OM changes following treatment.

The null hypothesis for this study is summarized as follows:

(H_0_) There is no difference in MT among treatment groups as assessed by MRI, CT, and histology.

## 2. Methods

### 2.1. Animals

28 chinchillas were obtained from Bowen's Chinchillas, LLC. All chinchillas were adult females, weighing between 500 and 700 grams each. Upon arriving at the Veterinary Medical Unit (VMU) at the Jerry L. Pettis Veterans Medical Center, they were placed in isolation and observed for one week. During this time, all chinchillas were screened for absence of OM by anesthetizing them with isoflurane (induction 3.5%, maintenance 2.5%) inhalation and evaluating each ear canal with a micro otoscope; they were all confirmed to be normal, healthy chinchillas, with absence of OM at baseline. All animal procedures were approved by the Institutional Animal Care and Use Committee at the Jerry L. Pettis Memorial Veterans Administration Medical Center.

The 28 chinchillas were divided into three treatment groups: 6 underwent treatment by vehicle control, 11 were treated with Ciprodex: ciprofloxacin 0.3%/dexamethasone 0.1% (DEX), and 11 received Cipro HC: ciprofloxacin 0.2%/hydrocortisone 1% (HC). The vehicle control was composed of the sterile, preserved suspension used to make Ciprodex and Cipro HC and was obtained from Alcon, Inc. This was selected as a control solution over other potential control substances, such as saline, because it remains as similar to the treatment groups, but without antibiotics or steroids present. The composition for the vehicle control includes benzalkonium chloride as a preservative, boric acid, sodium chloride, hydroxyethyl cellulose, tyloxapol, acetic acid, sodium acetate, edetate disodium, and purified water.

Ciprodex (ciprofloxacin 0.3% and dexamethasone 0.1%; NDC number 0065-8533-02) was obtained as a sterile otic suspension. It is a combination antibiotic (quinolone), steroid treatment commonly used by clinicians to treat acute otitis externa and acute OM following tympanostomy tube placement or with tympanic membrane perforation. Ciprodex confers broad-spectrum antibiotic protection with low adverse effects and minimal ototoxicity.

Cipro HC (ciprofloxacin 0.2% HCl and hydrocortisone 1%; NDC number 0065-8531-10) was also obtained as a sterile otic suspension. Similarly, it is a broad-spectrum antibiotic, steroid treatment.

Ciprodex and Cipro HC are registered trademarks of Bayer AG licensed to Alcon, Inc. and are available by prescription only. Their compositions are akin with the underlying difference being the anti-inflammatory corticosteroid: DEX versus HC.

### 2.2. Procedure

Chinchillas were injected via the superior bullae with 0.2 mL of their respective treatment. Two hours later, chinchillas were inoculated with 0.3 mL of *Salmonella enteric *lipopolysaccharide (LPS) and additional 0.2 mL treatments of respective treatments were injected into the inferior bullae at 24, 48, and 72 hrs after LPS inoculation. At 120 hrs postinoculation, chinchillas were euthanized using an 80 mg/kg ketamine intramuscular injection. Following animal sacrifice, chinchilla temporal bones were preserved in 10% formalin for 72 hrs, then washed in 10x PBS, and finally stored in 10x PBS, 0.1% azide at 3°C.

### 2.3. Radiographic Imaging

**MRI**

**Figure 1:**
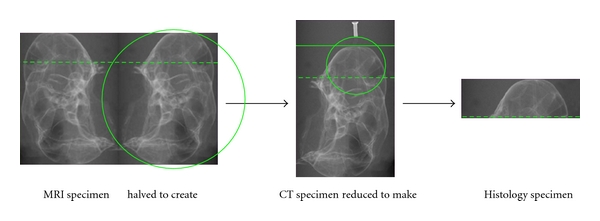
Description of specimen use. Each specimen began with a complete chinchilla skull, which was used for MRI imaging. Due to the size limitation of samples in the Micro-CT machine, chinchilla skulls were halved to accommodate CT imaging. Once imaging was completed, samples were further pared down to about one quarter of their original size for histology processing. Histology cassettes and molds limited the size of the sample.

**Figure 2:**
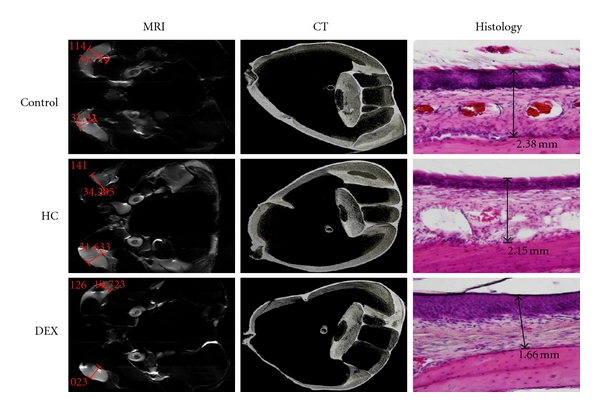
Imaging results compared by treatment group. Under the T2-weighted MRI produced images of MT that appear bright white for both right and left inferior bullae, whereas the dark space represents bone and/or air. The CT images were made for either the right or left bullae, with the bottom quadrant was targeted to match the MRI area that was measured. In the CT imaging, white represents moist MT. In the third column, histology images are presented, where MT demarcation is indicated by the arrow, and the bright pink outside the arrow bounds represents bone.

18 intact chinchilla temporal bones were taken to the Non-Invasive Imaging Laboratory at Loma Linda University and imaged using Brucker MRI 11.7T, which produced T-2 weighted images (field of view = 3 cm; slice thickness = 1 mm; interslice distance = 1 mm; number of slices = 30; number of echoes = 10; matrix = 256 × 256; resolution read = 0.0117 cm/pixel; repetition; time = 3593.9 ms; echo time = 10.2 ms; slice orientation =  sagittal) (Figure 1). Temporal bones were dried before imaging processes to reduce effects of water. MRI images were viewed and analyzed using Cheshire^©^ v4.3 software, where MT was measured using the ruler tool which produced measures in pixels. MT was then calculated into mm given that 1 pixel is equivalent to 0.117 mm. 10-11 MT measures were calculated per specimen for 36 chinchilla middle ears (18 temporal bones × 2 sides = 36 middle ear cavities), providing an *N* = 380 data points. Given that the MRI was T2 weighted, soft tissue MT in the inferior bullae appear bright white against empty space, bone, and residual water, which appears black (Figure 2).

**CT**

14 middle ear cavities that were MRI imaged were taken to the VMU at the Jerry L. Pettis Veterans Medical Center for imaging using Scanco vivaCT 40 (slice thickness = 10.5 microns; number of slices = 140). Due to the fact that the specimens were half of the MRI imaged samples (see Figure 1), a radiographic pin was inserted into the inferior bullae to facilitate CT imaging and maintain imaging consistency. Once again, specimen were dried before imaging processes to reduce effects of water. Images were analyzed using Scanco software. MT was measured in two ways: bone-to-mucus ratio, and mucosal volume. In the images, bones appears white and MT appears gray (Figure 2).

### 2.4. Histology

Following radiographic imaging, all chinchilla specimens were decalcified using 3 M EDTA over a four-week period with 3 EDTA changes. Following decalcification, specimens were further reduced to a quarter of their original size (see Figure 1) and placed in a paraffin processor overnight. Paraffin-embedded samples were sectioned at 15 microns over approximately the bottom-most 6 mm area of the inferior bullae. Sections were later stained by H&E and analyzed using an Olympus biological microscope with digital camera DP72. The DP72 computer software was used for image capture. MT measures were made in Adobe PDF Converter Professional off the images at unit length mm. In the histology images, decalcified bone is the lower pink layer, and MT is the middle and upper layers, appearing as a lighter pink/darker purple layer (Figure 2).

### 2.5. Statistics

SAS 9.1 was used to analyze significance of MT values and produce boxplots. Microsoft Excel was used to generate bar charts and tables.

## 3. Results

**Figure 3:**
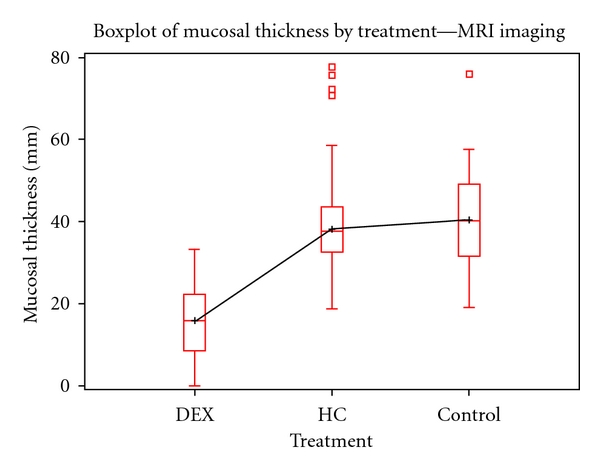
Boxplot of mucosal thickness by MRI Imaging. While there are some outliers present in the HC and control groups, the overall relationship of MT measures across treatment groups is statistically different, with DEX exhibiting significant MT reduction (*N* = 380, *α* = 0.05, *F* = 146.0861, *P*-value <0.0001). MRI MT measures were based in millimeters (mm).

MRI MT values were evaluated by treatment groups. The dataset consisted of *N* = 380 data points, with an overall and treatment group stratified distribution that appeared normal. ANOVA testing produced statistically different measures of MT among treatment groups (*F* = 146.0861, *P*-value <0.0001) (Figure 3). Post-hoc testing by Tukey revealed significant (*α* = 0.05) mean differences between treatment groups (Table 1). Box-plots and bar charts were created to represent the findings (Figures 3 and 6).

**Figure 4:**
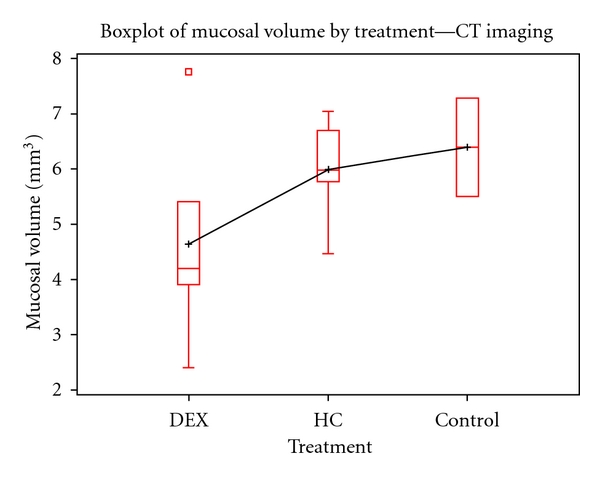
Boxplot of mucosal thickness by CT Imaging. Findings by CT also reveal that DEX treated chinchillas had less MT thickness when compared to the other groups. While the overall trend is comparable to the MRI and Histology results, the CT findings are not statistically significant at an *α* = 0.05 level due to the small sample size (*N* = 14, *χ* ^2^ = 0.9762, *P*-value  = 0.6138). CT measures of MT were based on mucosal volume (mm^3^).

CT MT values were also evaluated by treatment groups. The dataset was smaller, with an *N* = 14. The overall and group stratified distribution was grossly nonparametric. Kruskal-Wallis was used to analyze global differences across treatment groups but did not find any statistical significance when analyzing bone to mucosal thickness ratios (*χ* ^2^ = 1.2404, *P*-value = 0.5378) and total average calculated mucosal volume (*χ* ^2^ = 0.9762, *P*-value = 0.6138). Box-plots and bar charts were generated to show trends, but no additional post-hoc testing was conducted (Figures 4 and 7).

**Figure 5:**
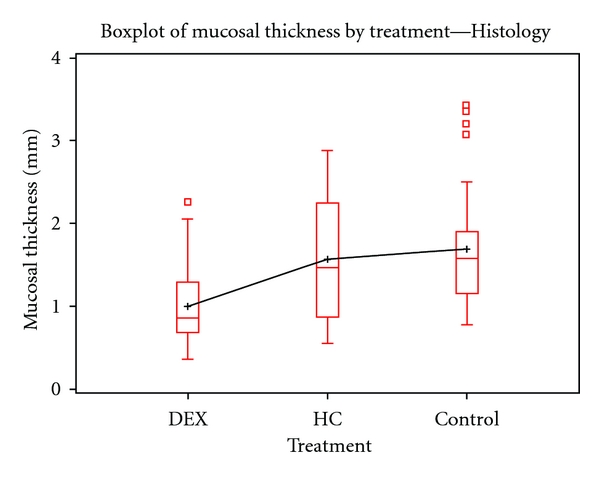
Boxplot of mucosal thickness by Histology. Histology results also show the reduced MT in the DEX-treated specimen compared to the other treatment groups. The findings were statistically significant (*N* = 194, *α* = 0.05. *χ* ^2^ = 40.5267, *P*-value < 0.0001). Histology measures of MT were made in millimeters (mm).

**Figure 6:**
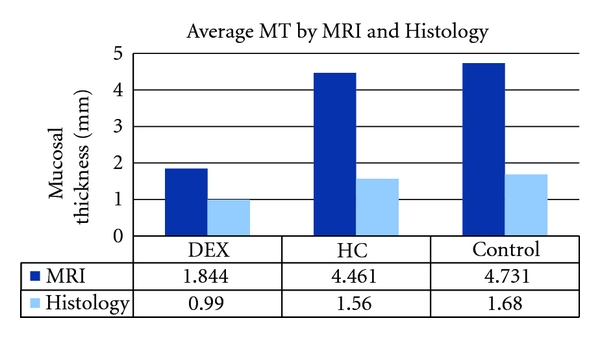
Measuring MT by MRI and Histology. This bar chart is a comparison of two methods for measuring MT, which show comparable findings. Both MRI and Histology methods reveal that DEX-treated chinchillas produced less mucosal thickness when compared to HC-treated and control groups. The disparity in the MT thickness is likely due to the specimen handling during histology, which requires use of various desiccating substances.

**Figure 7:**
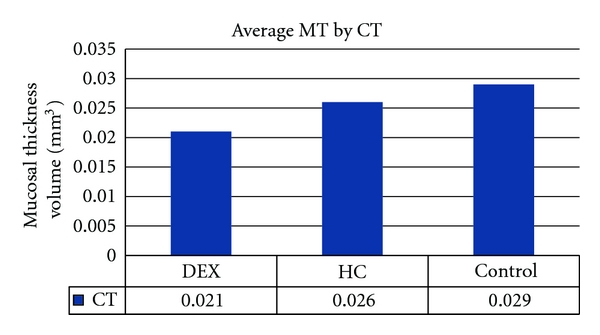
Measuring MT by CT. Measuring mucosal thickness by CT imaging produced measures of MT volume, which is why this bar chart stands alone. The findings, however, are similar to those observed in Figure 6, where DEX-treated chinchillas had less MT thickness compared to the other treatment groups.

Histology MT values were evaluated by treatment groups. The dataset consisted of *N* = 194 measures. The overall and group stratified distributions were nonnormative. Kruskal-Wallis was used to evaluate difference across treatment groups and revealed a significant finding (*χ* ^2^ = 40.5267, *P*-value <0.0001). Post-hoc testing with Tukey was conducted on the data and showed statistically significant differences (*α* = 0.05) between groups (Table 2). Box-plots and bar charts were made to show these differences graphically (Figures 5 and 6).

For both MRI and histology findings DEX-treated samples exhibited reduced MT compared to HC-treated and vehicle control subjects.

## 4. Discussion

Previous research investigating OM found that diagnosis and prognosis using anatomical imaging such as CT and MRI were preferred over nuclear medicine approaches. Furthermore, researchers found that by comparison to CT, MRI was better at exploring soft tissue changes over time [4]. Similarly, an investigation into chronic OM affecting the temporo-mandibular joint found the utility of MRI for imaging intracranial soft tissue masses and thrombosis of sinus tranversus to evaluate mastoiditis prior to therapeutic surgical techniques [5]. In this same study, investigators also used CT to reveal anatomical changes in bone and surrounding soft tissue mass but found that MRI provided more detailed analyses of infection severity by imaging soft tissue swelling [5]. Unofficial consensus favors MRI for its cost effectiveness, availability, high resolution, image detail, and absence of radioactivity [6].

 In this investigation, we found additional support for MRI use in imaging inflammation and specifically for tracking OM by means of MT. Our MRI data were easy to extract and analyze, producing an adequate sample size estimation of MT outcomes across groups. Our CT data were based on a relatively smaller sample size due to imaging demands and resources. CT results were somewhat harder to evaluate because of the high bone resolution and lower resolution mucosal layer. Given that our MRI and CT data were not exactly comparable, and previous reviews have commented on the inaccuracy of imaging OM [7], histology served as the point of reference. From histology results, we are able to confirm the utility of MRI for imaging MT as an inflammatory marker of OM. For future investigations, however, contrast enhanced MRI-imaging of *in-vivo*, inoculated specimen would provide greater detail of OM processes and changes over time; *in-vivo* imaging practices would parallel clinical manifestations more accurately.

This investigation also highlighted the promising role of DEX for improving inflammation of OM. MT measures across treatment groups using all analytic methods (MRI, CT, and histology) showed that DEX was more effective at decreasing MT. The use of DEX in combination with antibiotics seems to be a more effective treatment to reduce inflammation in the middle ear. MRI seems to be a better imaging method of determining stage and severity of acute or chronic OM.

## Figures and Tables

**Table 1:** Tukey's followup analysis for MRI measures of MT. MT measures across treatment grouping show that DEX was successful at reducing MT values when compared to both control and HC treated groups.

MT measures by MRI imaging *α* = 0.05		
Treatment comparison	Difference between means	Confidence limits
Control-HC	2.31	−1.199, 5.819
Control-DEX	24.678	21.169, 28.188
HC-Control	−2.31	−5.819, 1.199
HC-DEX	22.368	19.776, 24.96
DEX-Control	−24.678	−28.188, −21.169
DEX-HC	−22.368	−24.960, −19.776

**Table 2:** Tukey's followup analysis for Histology measures of MT. Histology confirmed MRI results for DEX efficacy at reducing MT. Though HC was effective at reducing MT when compared to control alone, the difference was not statistically significant. Only DEX significantly reduced MT compared to both HC and control treatment groups. The differences between means reported by histology are similar to those reported by MRI but seem smaller in magnitude. This may be attributed to histoprocesses that compromise tissue integrity (i.e., MT desiccation due to various chemical changes, other effects on MT resulting from paraffin impregnation, etc.). Differences in histology and MRI findings could also result from a difference in sample siza. *N* = 380 for MRI and *N* = 194 for histology.

MT measures by Histology *α* = 0.05		
Treatment comparison	Difference between means	Confidence limits
Control-HC	0.121	−0.17271, 0.415
Control-DEX	0.690	0.40969, 0.971
HC-Control	−0.121	-0.41537, 0.173
HC-DEX	0.569	0.33475, 0.803
DEX-Control	−0.690	−0.97086, −0.410
DEX-HC	−0.569	−0.80314, −0.335
